# A case study of organisational cultural competence in mental healthcare

**DOI:** 10.1186/1472-6963-11-218

**Published:** 2011-09-15

**Authors:** Jean Adamson, Nasir Warfa, Kamaldeep Bhui

**Affiliations:** 1 The Diversity Academy, Woodford Green, Essex, IG8 8GJ, England, UK; 2 Centre for Psychiatry, Wolfson Institute of Preventive Medicine, Barts and The London School of Medicine & Dentistry, Queen Mary University of London, Old Anatomy Building, Charterhouse Square, London, EC1M 6BQ, England, UK

**Keywords:** organisational cultural competence, mental health

## Abstract

**Background:**

Ensuring Cultural Competence (CC) in health care is a mechanism to deliver culturally appropriate care and optimise recovery. In policies that promote cultural competence, the training of mental health practitioners is a key component of a culturally competent organisation. This study examines staff perceptions of CC and the integration of CC principles in a mental healthcare organisation. The purpose is to show interactions between organisational and individual processes that help or hinder recovery orientated services.

**Methods:**

We carried out a case study of a large mental health provider using a cultural competence needs analysis. We used structured and semi-structured questionnaires to explore the perceptions of healthcare professionals located in one of the most ethnically and culturally diverse areas of England, its capital city London.

**Results:**

There was some evidence that clinical staff were engaged in culturally competent activities. We found a growing awareness of cultural competence amongst staff in general, and many had attended training. However, strategic plans and procedures that promote cultural competence tended to not be well communicated to all frontline staff; whilst there was little understanding at corporate level of culturally competent clinical practices. The provider organisation had commenced a targeted recruitment campaign to recruit staff from under-represented ethnic groups and it developed collaborative working patterns with service users.

**Conclusion:**

There is evidence to show tentative steps towards building cultural competence in the organisation. However, further work is needed to embed cultural competence principles and practices at all levels of the organisation, for example, by introducing monitoring systems that enable organisations to benchmark their performance as a culturally capable organisation.

## Background

The relationship between ethnicity and mental health has been the focus of much debate and dispute for many years. International research has shown that Black and Minority Ethnic (BME) communities fear and mistrust mental health services, and that BME groups, as they are called in UK policy, feel alienated and generally misunderstood (Mohan et al. 2006 [1]; Bhui et al. 2003 [2]; Leese et al. 2006 [3]; Fearon et al. 2006 [4]; Warfa et al 2006*a *[5]; Warfa et al 2006*b *[6]). This literature also shows that patients from ethnic minority backgrounds are less likely to attend their GP, and more likely to be placed on compulsory treatment orders. Another area of disparity has manifested itself through pathways into mental health care; service users from some specific ethnic groups are more likely to be admitted via the criminal justice system. A large number of these studies have suggested that action should follow demonstrable ethnic differences in relation to diagnosis, rates of inpatient admissions, care pathways and compulsory detention. Another group of studies examines racial discrimination and mental illness (e.g., Bhui et al. 2005 [7]; Karlsen et al. 2005 [8]; Bhugra & Ayonrinde, 2001 [9]). A smaller set of studies has focused on acculturation and mental illness. (e.g., Bhui et al. 2005 [10]; Berry, 1988 [11], 1997 [12]). However, very few studies have investigated the issue of organisational CC, with the exception of Siegel et al. (2000 [13], 2003 [14]) and Stork et al. (2001 [15]) in the US. From these limited studies, there is some evidence to support the notion that there is a relationship between the CC of health practitioners and the CC of organisations. For example, Organisations that incorporate CC into their strategic planning and operational policies are more likely to develop health practitioners and leaders that are culturally competent (Paez et al. 2007 [16]). In the UK, the concept of cultural competence has developed from within the diversity framework and was initially applied within the US health and social care systems. It is suggested that the term came into use because it fits with work-based frameworks for competencies in a number of areas, an approach that has been used to train and maintain a competent professional workforce.

Cultural Competence begins with understanding the strengths and weaknesses of the healthcare organisation and the unique needs of the population being served. Cross et al. (1989 [17]) defined cultural competency as *"a set of congruent behaviours, attitudes, and policies that come together in a system, agency or among professionals and enables that system, agency or those professionals to work effectively in cross cultural situations"*. In other words, the concept of cultural competence (CC) has evolved as multicultural societies have grown and mental health services are required to respond to a range of cultural needs from the communities that they serve. CC is based on the premise that culturally appropriate care aids the recovery process; therefore, a skilled workforce has become central to the development of cultural competence in organisations.

There are numerous models of cultural competence. Davis (1997 [18]) postulated that at an operational level cultural competence is the ability to integrate knowledge about individuals and groups into specific standards, policies, practices and attitudes, which are used to improve the quality of health care and ultimately produce better outcomes. Cross et al's (1989) model identifies a continuum of cultural competence from cultural proficiency at one end through to cultural destructiveness at the other. It is a process of increasing proficiency gained from informal and formal cross-cultural experiences rather than an endpoint that is achieved. Culturally competent organisations actively design and implement services that are relevant to the needs of their service users. Within the organisational context, CC can be sought at various levels; including direct clinical care, operational management and corporate managerial levels. In this study we address all of these levels by taking into account the perceptions of staff across the organisation.

In the UK, Black and Minority Ethnic (BME) groups have largely encompassed those from African, Caribbean and Asian origins as well as 'racialised' white people, for example, East Europeans, the Irish and Gypsy and travelling communities. Research has shown that BME groups feel alienated, and generally misunderstood (Breaking the Circles of Fear, Sainsbury Centre for Mental Health 2002 [19]). The campaign for improving mental health services for BME populations emerged from three key UK government publications: *Inside Outside (NIMHE 2003 *[20]*), Delivering Race Equality (DoH 2005 *[21]*) and Race Equality Training in Mental Health Services in England *(DoH 1999 [22]). *Inside Outside (NIMHE 2003) *describes a programme for reform of mental health services for (BME) communities in England. It builds on standards set out in the Mental Health National Service Framework (MHNSF) and the NHS Plan. The MHNSF (DoH 1999 [23]) recognised that services were not adequately meeting the needs of BME service users; whilst the NHS Plan sets out a blueprint for high quality care in the twenty first century. *Inside Outside's *change initiatives focussed on actions both *'inside'- *within the services- and *'outside' - *within the communities; cognisant of the need for collaboration between BME communities, voluntary and statutory sector services to develop services that are appropriate and responsive to the needs of minority ethnic communities. Two key components of the strategic plan were to reduce and eliminate ethnic inequalities in mental health service experience and outcome and to develop the cultural competence of mental health services through education and training.

Since much of the research on cultural competence has taken place in North America, it is reasonable to question the relevance of findings to the UK setting. There are apparent parallels evidenced in the increasing diversity of the population; the similarity in ethnic health inequalities and the growing demand for cultural competence in health care systems. In the US and UK, ethnic and racial disparities in health care form the basis for actions to develop culturally competent practice; in recent years these issues have come to the forefront of healthcare policy and research, not least as these inequalities persist, indeed in the UK the ethnic disparities in compulsory admission are sustained such that the Care Quality Commission (a regulating body) has decided to stop collecting the data and reporting on these through other data sources five yearly rather than annually. This is ironic given the lack of progress, but perhaps belies the underlying organisational narratives and a failure of achieving organisational cultural competence. These inequities are well documented in the literature, some of which is reviewed in this paper and reported in a systematic review (Bhui et al. 2007 [24]). All of the studies in the systematic review, with the exception of one, were North American; the nature of health inequalities experienced in health care, nevertheless, are not dissimilar from those experienced in the UK, although there are some fundamental differences between the UK and US healthcare systems and the specific histories and cultural influences and life events that lead to social exclusion and mental illness.

The context in which health care is delivered differs in the UK due to the welfare system and in particular the NHS; which is free at the point of delivery. In the US, healthcare is largely privatised and 'managed care' functions to gate keep healthcare services; hence healthcare is related not only to patients' needs but also affordability, a pattern which is becoming evident in the UK as the costs of NHS care are rising. So universally, health inequalities are related to socioeconomic status and ethnicity; however, health care will inevitably be influenced by the health care system. In this context, given the lack of studies from the UK and studies of organisations, this paper reports on a three level cultural competence needs analysis that examines whether CC principles are embedded in organisational systems.

## Methods

### Location

Mixed methods were used to conduct a cultural competence needs analysis of a large London based NHS provider organisation (called a NHS Trust). The areas served by the NHS Trust are the most culturally diverse and deprived areas in England and therefore provide significant challenges for the provision of mental health services. The provider organisation's local services cater for a multi-ethnic population of 710,000 and forensic services are provided to a population of 1.5 million.

### Measures of Cultural Competence

Culturally competent practice is not limited to individual professional practice; rather there is an organisational component that comes with a statutory obligation to ensure that its workforce and service users can expect to be treated in a way that is appropriate and responsive to their cultural needs. Such measures are not instead of existing standards of care quality but in addition to in order to eliminate inequalities. Siegel et al., (2000) developed a conceptual framework with interacting domains of CC; six domains of CC were identified: needs assessment, information exchange, services, human resources, policies/procedures and CC outcomes. In a follow up study Siegel et al., (2003) benchmarked a selection of these performance measures in several mental health facilities. The six CC domains were utilised in this study to develop the data collection tools. Prior to the study, the questionnaire was piloted within the provider organisation; which brought a helpful response and revisions were made to the format and style of the questionnaire. Using these tools, cultural competency was measured across three levels of the organisational structure: corporate, operational and direct care. Culturally competent activities in these domains were hypothesised to lead to positive outcomes for multicultural and multiracial clients. The domains are defined below:

1. Needs Assessment - availability of **information on characteristics of population in treatment**, i.e. demography, socioeconomic status, languages spoken, literacy levels, cultural beliefs & practices.

2. Information Exchange - **Exchange of information between local communities & the Trust**, i.e. concerns of multicultural and multiracial groups and information from the organisation to the community re: services offered.

3. Human Resources - CC **training; recruitment of cultures representative of the community **and who speak the languages of the target population of the area. KSF should reflect adherence to CC principles.

4. Services - Service users and carers need to be **involved in the development of services**. Services are responsive to cultural needs.

5. Policies/Procedures - Trust wide **CC plan should be formulated with representation from local community **then disseminated to all sites (inpatient & community services). Information systems include cultural and ethnic characteristics.

6. Cultural Competence Outcomes - **Desirable outcomes **for service users are evidenced by clinical change, increased social functioning and recovery.

The provider organisation was divided into three levels using the following definitions:

#### Corporate level

The mental health authority that has organised and administers the care system. It comprises key functions of the organisation i.e. *Information Technology, Human Resources etc*.

#### Operational level

Those employed by the mental health authority responsible for operations and service delivery i.e. *Senior managers based on hospital sites and in the community setting*.

#### Direct Care Level

Those employed by the mental health authority responsible for provision of direct care i.e. *nurses, doctors*.

### Procedure for authorisation to undertake staff survey

The provider organisation's ethics committee approved this project subject to a few amendments. Concerns raised by the ethics committee related to the use of Americanised terminology and the perceived sensitivity of some questions. Necessary amendments were made, and prior to the survey, the audit and evaluation tool was piloted for face validity and feedback obtained on style, format and content.

### The Sample

Three samples were selected, one for each of the corporate, operational and direct care levels. The direct care level sample was selected using the stratified sampling method, and comprised 336 clinical staff of which two thirds were nurses. Others included doctors, occupational therapists, psychologists, social therapists and healthcare support workers. In addition, a purposive sample of 36 staff was taken for the operational and corporate level participants. Thirty operational level staff selected included directors and senior nurse managers. At corporate level, six department heads and directors were selected representing key functions of the Trust.

### Data Collection

For this study, two questionnaires were devised based on the six domains; one questionnaire was used in a postal survey, at operational and direct care levels. The second questionnaire was used at corporate level and asked the same questions, but in an open-ended style to allow exploration of issues for the semi-structured interviews. A participant information sheet was incorporated into the tool outlining the participant's right to withdraw and ethical considerations such as confidentiality and anonymity. For consistency and clarity in the postal survey, the concept of 'cultural competence' was defined and explained before the questions. Cultural Competence was defined as *"the set of behaviours, attitudes, skills, policies and procedures that come together in a system, agency, or individuals to enable mental health care givers to work effectively and efficiently in multicultural situations"*.

The aim of the evaluation at the corporate level was to elicit understanding of CC and organisational engagement, and given the methods were open-ended interviews, a standard CC definition was not deemed necessary as answers were to be qualified by respondents and so intended meaning could be checked at interview. Respondents were asked to report their ethnicity, job title and work location.

### Analysis

Excel software was used to calculate descriptive statistics. The *framework approach *(Miles & Huberman 1984) was used to analyse the semi-structured interviews. This approach is based on matrix based methods of analysis, and it is both inductive and deductive in nature. The analytic process is linked to the aims and objectives of the study; whilst also being rooted in the original accounts and observations of the people studied. This method was chosen for its transparency and procedures that are replicable and valuable in policy and practice research. The researcher acquired an intimate knowledge of the raw data. The process of *familiarisation *with the data began on conducting the semi-structured interviews. The collection of data and the analysis of data were not entirely discrete activities, since themes began to emerge during the data collection phase. The analysis moved iteratively through stages of data management, description and explanation. Several themes were identified and then numbered and named using short descriptive text. Distilled summaries were taken from the original data and charted onto an Excel spreadsheet, according to the appropriate part of the thematic framework to which they related. The process of mapping and interpretation of the data was influenced by the original research objectives as well as the themes that emerged from the data itself.

## Results

Seventy-three of 336 staff from the direct care sample responded to the postal questionnaire. Two thirds of the respondents were nurses, mostly of Black African ethnic origin; this was in accord with the proportional of Black African nurses employed in the Trust. White British and Black Africans represented 82% of all respondents (Table 1). Fourteen of 30 staff from the operational sample responded to the questionnaire, almost half were White British and the remainder were from BME groups (Table 2).

**Table 1:** Direct care level respondent characteristics, *n *= 73

Characteristic	*n *(%)
Gender	
*Female*	43 (58)
*Male*	30 (42)

Ethnicity	
*White British*	27 (37)
*Black Caribbean*	2 (3)
*Black African*	33 (45)
*Asian*	7 (10)
*Indo Caribbean*	1 (1)
*Mixed Race*	3 (4)

Discipline	
*Nurse*	49 (68)
*Doctor*	6 (8)
*Psychologist*	3 (4)
*Occupational therapist*	4 (5)
*Social worker*	3 (4)
*Social therapist*	3 (4)
*Healthcare support worker*	5 (7)

**Table 2:** Operational level respondent characteristics, *n *= 14

Characteristic	*n *(%)
Gender	
*Female*	8 (57)
*Male*	6 (43)

Ethnicity	
*White British*	6 (43)
*Black Caribbean*	3 (21)
*Black African*	3 (21)
*Asian*	1 (7.5)
*Indo Caribbean *	1 (7.5)
*Mixed Race*	0 (0)

Discipline	
*Manager*	8 (57)
*Modern Matron*	5 (36)
*Borough Nurse*	1 (7)

### Operational & Direct Care Levels

There was a tendency for operational and direct care level staff to agree in their response to questions pertaining to frontline CC practices. However, they were divided on issues such as whether someone is appointed with responsibility for CC in the organisation and if reference is made to CC in the Trust mission statement (Table 3). This may be an indication that messages about CC are not filtering through to direct care staff.

**Table 3:** Questionnaire Response at Direct Care & Operational Levels

Direct care level n = 73	Operational level n = 14	
Question	Direct care	Operational

	*yes (%)*	*yes (%)*

*Cultural/spiritual needs assessed*	79.4	78.5

*Services advertised*	57.5	42.8

*Service info available in languages*	65.7	50.0

*Cultural competence training available*	72.6	71.4

*Cultural competence training attended*	35.6	35.7

*Staff representative of communities*	67.1	42.8

*Performance appraisal reflect cultural competence*	61.6	57.1

*Engaging of service users in service planning*	56.1	57.1

*Person responsible for cultural competence*	24.6	57.1

*Language assistance available*	76.7	78.5

*Bilingual staff members in team*	94.5	100

*Cultural competence in Trust mission statement*	67.1	92.8

*Direct care level = clinical staff*		
*Operational level = senior managers*		

**Example of question asked: ***Are the cultural/spiritual needs of the service user assessed during the initial screening/intake into the service?*

### Corporate Level

A list of emerging themes were noted and then categorised into sub-groups and coded for easy retrieval. They were further refined to the point where four broad themes were conceptualised under the following headings, conveying the key ideas and forming the *thematic framework*:

#### Perceptions of cultural competence

Participants had a tendency to describe cultural competence in terms of understanding differences in the cultural context, and the ability to change and adapt clinical practice to meet the client's cultural needs. The need to be aware of one's own prejudices and biases was seen as prerequisite for that change to take place. Views expressed supported the idea that cultural competence training is not transferable; but rather, that it should be contextual and relevant to the type of service being delivered. Collectively, participant discourse mainly focused on the individual practitioner's responsibility for cultural competence practice, with the organisational responsibility largely ignored. The provider organisation had recently implemented a race equality cultural competence (RECC) programme; which is mandatory for all clinical staff. This is in addition to the equality & diversity training that all new staff received on the induction programme.

#### Equality in the workforce

Under-representation of BME staff in senior management and at board level, in particular, is problematic. Targeted recruitment of local communities was identified as an initiative to attract greater numbers of ethnic minorities; however it was also recognised that the campaign was designed to fill lower graded posts in the organisation rather than senior management positions. Poor quality data on ethnicity of the workforce had weakened the organisation's ability to understand how issues of equality impact on its staff. Other ongoing initiatives included training of career advisors as a resource for employees, and the promotion of programmes for BME staff seeking career succession into senior management and director level positions.

#### Patient Information

The provider organisation owns a substantial amount of service user data collected through patient assessment systems. There is inconsistency between data that has been collected on paper and data that is collected electronically, with little understanding about types of information held on the patient information database. Disparate accounts were expressed about the utilisation of patient data; although it was apparent that participants' views about utilisation of data were consistent with statutory legislation about improving ethnicity data. The provider organisation had not made comparisons of the representativeness of BME inpatients in relation to local population statistics, although relevant data is available for such analysis to take place. There was evidence though, that steps had been taken to acquire demographic data to support service planning. The Trust had recently commissioned a report to map the age, ethnicity and religion of its local population with predictive patterns for the coming years.

#### Race Equality in Organisations

Delivery of Race Equality encapsulates a multitude of activities that emanate from statute, and which are pivotal for the reform of mental health services. Many change initiatives were identified by participants. Less was known about the duties levied by race equality legislation. On the topic of race equality there was a tendency to defer to staff that lead on cultural competence. Accountability for race equality was not seen as a shared responsibility, but was articulated as the domain of specialist staff, conveying a lack of ownership and the location of responsibility for any action to a small group of staff often in relatively junior positions.

## Discussion

The findings of this study indicate that the provider organisation has taken several definite steps towards building the cultural competence of the organisation. Issues around recruitment & retention, improving data, engaging faith communities, training, challenging stigma, impact assessing policies and systems were ongoing. Structures were in place to facilitate engagement with multicultural and multiracial communities. Although the provider organisation had been slow to embrace the cultural competence agenda, and had yet to embed cultural competence principles at all levels of the organisation; our data suggest that the cultural relevance of services had moved up on the agenda.

Cultural Competence should not only reflect competence in delivering interventions to culturally diverse populations but also the development of interventions to engage and help recovery where existing models of treatment are ineffective or lead to non-adherence. Given the complexity of care services, cultural competent practice must be effective across organisational systems in general. It is argued that CC ought to be integral to policy, administrative practices and service delivery, and should be informed through service user and community involvement. Whilst CC training has focused on cultural difference and encompasses a wide range of activities designed to improve cultural awareness, knowledge and understanding; fewer CC activities highlight the adaptation of specific clinical practices or procedures for improved take up and recovery; and few studies investigate cultural considerations in prescribing practice.

From our study, commonly expressed views of CC comprise a discourse that focuses largely on diversity, cultural awareness and cultural sensitivity. Cultural competence training was described in terms of changing of attitudes & behaviour, self-reflection, acquisition of skills, knowledge and increasing awareness. Culturally competent outcomes could not be formally identified anywhere within the Trust; nor were performance measures used or applied by commissioners or providers or regulators. The NHS staff appraisal system offers a competency based assessment of staff performance. Although one of its core dimensions is Equality and Diversity, respondents observed that there is insufficient guidance on what skills/knowledge constitutes CC.

Overall, it was apparent that there was greater convergence in the views of corporate and operational level staff than corporate and direct care clinical staff; notwithstanding corporate level staff were less well informed about cultural competence initiatives happening in clinical practice. For example, most corporate managers interviewed demonstrated scant awareness of culturally appropriate services or their provision in the provider organisation.

The Trust sets out its vision in the annual plan and it appears on their website. Strategic objectives outline the development of a culturally capable workforce, the need for culturally sensitive services and recruitment of staff that are locally representative. However, the organisation's Board of Directors and its Senior Management Team is not representative of the cultural and racial diversity of local communities. A particular form of cultural competence (RECC) training is mandatory for all clinical staff; however the same does not apply to senior managers and directors. The nature of this mandatory training does not address clinical practice, but issues of race awareness and communication. It has been suggested that CC training may not adequately meet the needs of senior managers and directors; indeed, recent reports indicate that managers would benefit from training with an interpersonal focus on managing BME staff.

Corporate managers described CC in the context of understanding cultural differences and shared a narrow interpretation of cultural competence. This finding is consistent with literature that shows that cultural competence training has tended to focus on understanding difference, rather than reducing inequalities that affect BME groups. Little attention was paid to how culture impacts upon clinical presentation and the acceptability of specific interventions. Consensus indicated that CC principles were not adequately represented in the staff performance management framework. The local academic provider established an MSc in Transcultural Mental Healthcare to support workforce development (http://www.wolfson.qmul.ac.uk/psychiatry/courses/tmh[25]), a cultural consultation club as a resource for staff, and more recently, a cultural consultation service for work with narratives of recovery among commissioners, providers, and staff. (http://www.culturalconsultation.org.uk[26]).

Although this is a case study, there are broader implications of these findings for other provider organisations in the UK; especially with regards to training, policy making and strategic planning in general. CC Training should form part of a wider framework for reducing race inequality and address the needs of the organisation and its staff. It is not enough for direct care practitioners to undergo training; those who administer the provider organisation should educate itself on the dynamics of difference and develop cultural knowledge in order to make decisions that are not ethnocentric. Strategically, it is essential to develop a CC plan and define CC outcomes so that organisational self-assessment can take place. The policy making process should assess the impact on diverse communities. These strategic activities require input from local communities if services are to reflect diverse needs of service users. Accordingly, future research can build our understanding of CC further by eliciting the service user perspective.

### Strengths and limitations of study

The use of mixed methods in this study has produced a greater yield over and above a qualitative study or quantitative study undertaken independently. The interviews with corporate managers in the organisation allowed for richer and deeper insights into attitudes towards cultural competence; and most crucially offered insight into the sense of ownership felt by influential individuals.

The meaning attached to this study needs to be understood in the national context. There has been resistance to delivering race equality reforms; much criticism has been levelled at government agencies including mental health provider organisations around the UK (Bhui K, Ascoli M, Nuamh O: The Place of Race and Racism in Cultural Competence; the English Experience about the Narratives of Evidence and Arguments, submitted). Other factors that may introduce potential biases include the use of selective sampling; the local focus of the study, and the limited response rate to the study questionnaire.

Since there were no validated CC assessment tools in use in the UK, the questionnaire used in this study was modelled on performance measures developed in North America where health care systems are characteristically different from the UK; this in itself imposes a limitation in terms of the validity of the questionnaire for a UK study.

## Conclusion

Change occurs in a complex interplay between practice and policy set in the context of narratives of change and the culture of the system. Cultural competence is a developmental process and takes time, commitment and sustained effort at each level of the organisation. In recent years, the provider organisation has progressed in putting in place infrastructure to enhance its cultural competence; however, there is no strategy or policy to build cultural competence or dedicated strategic planning in the organisation, despite the local population being one of the most diverse in the country. Culturally inappropriate care practices and perceptions of injustice and inequity are likely to continue in the absence of a whole systems approach.

## Competing interests

'KB is Director MSc Transcultural Mental Healthcare, MSc Psychological Therapies, Director of Cultural Consultation Service in Tower Hamlets London.'

NW is senior lecturer, deputy director and MSc programme coordinator

The MSc programme has received support from the Local Mental Health Trust

NW is on the executive committee of the Cultural Consultation Club

## Authors' contributions

JA undertook the service evaluation supervised by KB and NW. All authors contributed to the consecutive versions of the paper. All authors have read and approved the final manuscript.

## Pre-publication history

The pre-publication history for this paper can be accessed here:

http://www.biomedcentral.com/1472-6963/11/218/prepub

## References

[B1] Mohan R Mone P Szmutler G Ethnic differences in mental health service use among patients with psychotic disorders' Social Psychiatry, Psychiatric Epidemiology 2006 41 771 776 10.1007/s00127-006-0094-7 16847582

[B2] Bhui K Stansfeld S Hull S Ethnic variations in pathways to and use of specialist mental health services in the UK British Journal of Psychiatry 2003 182 105 116 10.1192/bjp.182.2.105 12562737

[B3] Leese M Thornicroft G Shaw J Ethnic differences among patients in high security psychiatric hospitals in England British Journal of Psychiatry 2006 388 380 385 10.1192/bjp.188.4.380 16582066

[B4] Fearon P Kirkbride J Morgan C Incidence of schizophrenia and other psychoses in ethnic minority groups: Results from the MRC AESOP study Psychological Medicine 2006 36 1541 1550 10.1017/S0033291706008774 16938150

[B5] Warfa N Bhui K Craig T Mohamud S Stansfeld S McCrone T Thornicroft G Curtis S Post-migration residential mobility, mental health and health service utilization among Somali refugees in the UK: A Qualitative Study Health & Place 2006 12 503 515 10.1016/j.healthplace.2005.08.016 16203171

[B6] Warfa N Bhui K Phillips K Nandy K Griffiths S Comparison of live events, substance misuse, service use and mental illness among African-Caribbean, Black African and White British men in East London: a qualitative study Diversity in Health and Social Care 2006 3 111 21

[B7] Bhui K Stansfeld S McKenzie K Racial/ethnic discrimination and common mental disorders among workers: Findings from the EMPIRIC study of ethnic minority groups in the UK' American Journal of Public Health 2005 95 496 501 10.2105/AJPH.2003.033274 15727983 PMC1449208

[B8] Karlsen S Nazroo J McKenzie K Racism, psychosis and common mental disorder among ethnic minority groups in England Psychological Medicine 2005 35 1795 1803 10.1017/S0033291705005830 16194282

[B9] Bhugra D Ayonrinde O Racism, racial life events and mental ill health Advances in Psychiatric Treatment 2001 7 343 349 10.1192/apt.7.5.343

[B10] Bhui K Lawrence A Klineberg E Acculturation and health status among African-Caribbean, Bangladeshi and White British adolescents Social Psychiatry, Psychiatric Epidemiology 2005 40 259 266 10.1007/s00127-005-0890-5 15834776

[B11] Berry J Kim U Acculturation and mental health Health and Transcultural Psychology: Towards Applications 1988 Dasen PR, Berry JW, Sartorius N 207 238

[B12] Berry J Immigration, acculturation and adaptation Applied Psychology: An International Review 1997 46 5 68

[B13] Siegel C Davis Chambers E Haugland G Performance Measures of Cultural Competency in Mental Health Organisations Administration and Policy in Mental Health 2000 28 91 106 10.1023/A:1026603406481 11194126

[B14] Siegel C Haugland Davis Chambers E Performance measures and their benchmarks for assessing organisational cultural competency in behavioural health care service delivery Administration and Policy in Mental Health 2003 31 41 170 10.1023/b:apih.0000003019.97009.15 14756197

[B15] Stork E Scholle S Greeno C Copeland VC Kelleher, Monitoring and Enforcing Cultural Competence in Medicaid Managed Behavioural Health Care Mental Health Service Research 2001 3 3 169 177 10.1023/A:1011575632212 11718208

[B16] Paez K Allen J Carson K Cooper L Provider and Clinic Cultural Competence in a Primary Care Setting Social Science & Medicine 2007 5 1204 16 18164114 10.1016/j.socscimed.2007.11.027 PMC2426909

[B17] Cross TL Bazron BJ Dennis KW Isaacs MR Towards a culturally competent system of care A Monograph on Effective Services for Minority Children Who Are Severely Emotionally Disturbed 1989 1 Washington DC: Georgetown University Child Development Centre

[B18] Texas Department of Health National Maternal & Child Health Resource Center on Cultural Competence (1997)

[B19] Sainsbury Centre for Mental Health Breaking the Circles of Fear 2002

[B20] NIMHE Inside Outside: Improving mental health services for black and minority ethnic communities in England 2003 Leeds: National Institute for Mental Health in England

[B21] DH Delivering Race Equality in Mental Health Care. An action plan for reform inside and outside services and the Government's response to the independent inquiry into the death of David Bennett 2005 London: Department of Health

[B22] Sainsbury Centre for Mental Health Race Equality Training in Mental Health Services in England 1999

[B23] DH National Service Framework for Mental Health: Modern standards and service models 1999 London: Department of Health

[B24] Bhui K Warfa N Edonya P Cultural competence in mental health care: A review of model evaluations BMC Health Services Research 2007 7 15 10.1186/1472-6963-7-15 17266765 PMC1800843

[B25] MSc Transcultural Mental Healthcare http://www.wolfson.qmul.ac.uk/psychiatry/courses/tmh

[B26] Cultural Consultation Service http://www.culturalconsultation.org.uk

